# Two-step transjejunal biliary drainage using a colonoscope and an oblique-viewing linear-array echoendoscope after pancreatoduodenectomy

**DOI:** 10.1055/a-2966-7087

**Published:** 2026-10-06

**Authors:** Ryo Sugiura, Masaki Kuwatani, Kazumichi Kawakubo, Katsuma Nakajima, Issei Ashida, Naoya Sakamoto

**Affiliations:** 1 Department of Gastroenterology and Hepatology 163693 Hokkaido University HospitalSapporoJapan

In patients with surgically altered anatomy, forward-viewing echoendoscopes have recently been reported as a useful option for endoscopic ultrasound–guided biliary drainage (EUS-BD). 1​ 2​ ​3​ However, their availability may be limited. We present a case of successful transjejunal EUS-BD for complete obstruction of the posterior bile duct after pancreatoduodenectomy using an oblique-viewing linear-array echoendoscope.

A 68-year-old woman underwent subtotal stomach-preserving pancreaticoduodenectomy for a pancreatic head intraductal papillary mucinous adenoma. Preoperative cholangiography showed a lower-level confluence of the right posterior sectoral bile duct ( Fig. 1 ), which was inadvertently divided and sutured during surgery, resulting in complete obstruction ( Fig. 2 ). After developing cholangitis, percutaneous transhepatic biliary drainage was performed. As internal drainage was desired, endoscopic intervention was attempted after guidewire passage from the percutaneous route into the jejunum had failed. A colonoscope (PCF-H290ZI; Olympus Medical Systems, Tokyo, Japan) was advanced into the deep afferent limb ( Fig. 3a ). A 0.035-inch guidewire was placed in the jejunum, after which the colonoscope was withdrawn ( Fig. 3b ). An oblique-viewing linear-array echoendoscope (GF-UCT260; Olympus Medical Systems) was carefully advanced by following the guidewire as a landmark ( Fig. 4a ). The echoendoscope reached the jejunum adjacent to the obstructed bile duct ( Fig. 4b ), which was visualized by EUS ( Fig. 4c ). The bile duct was punctured with a 19-gauge needle, and contrast injection confirmed the isolated posterior bile duct ( Fig. 5a ). After tract dilation, a 7-Fr, 10-cm plastic stent was placed between the posterior bile duct and the jejunum ( Video 1 , Fig. 5b–d ).

**Fig. 1:**
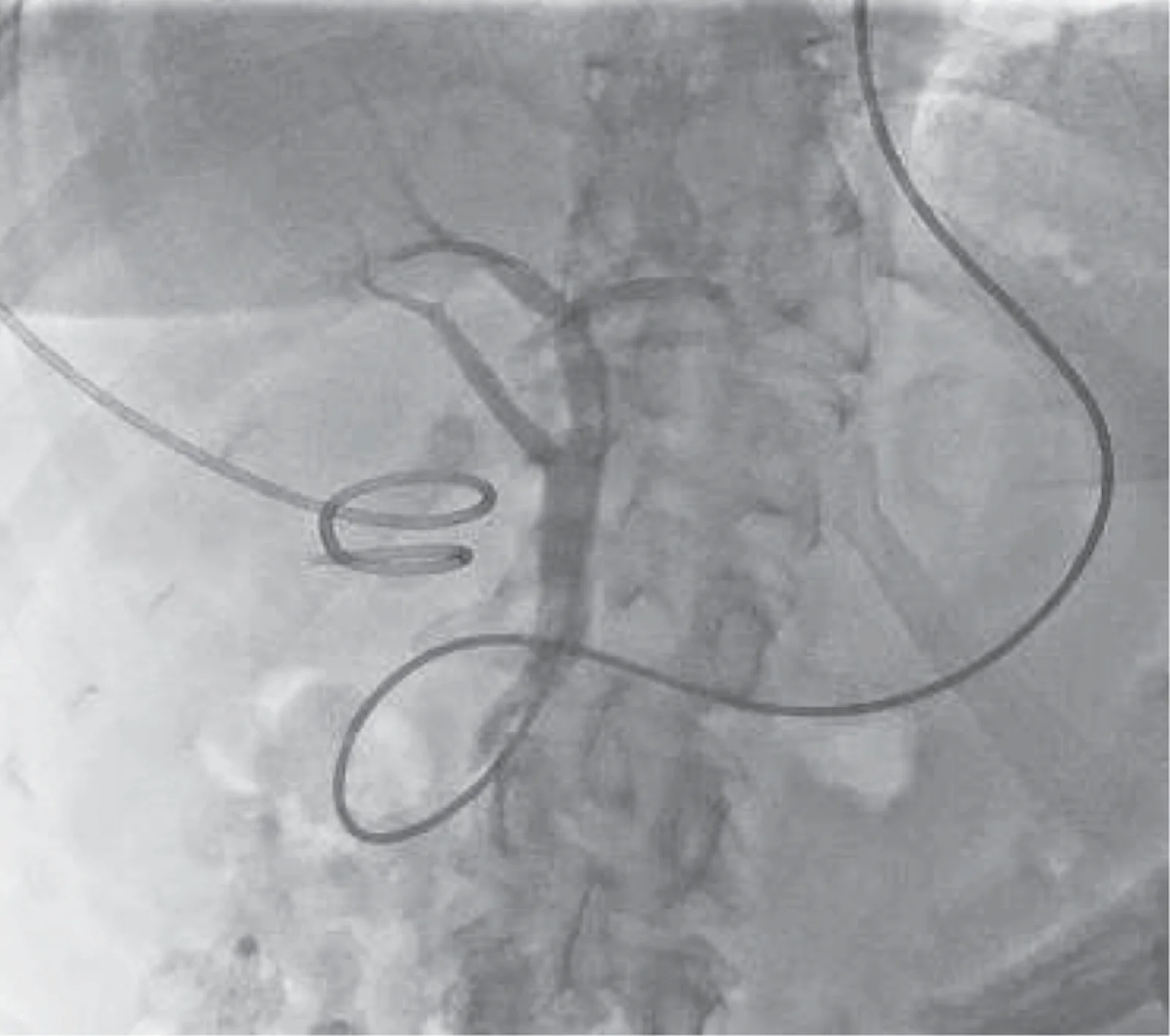
Cholangiography through a preoperatively placed nasobiliary tube showing the lower-level confluence of the right posterior sectoral bile duct.

**Fig. 2:**
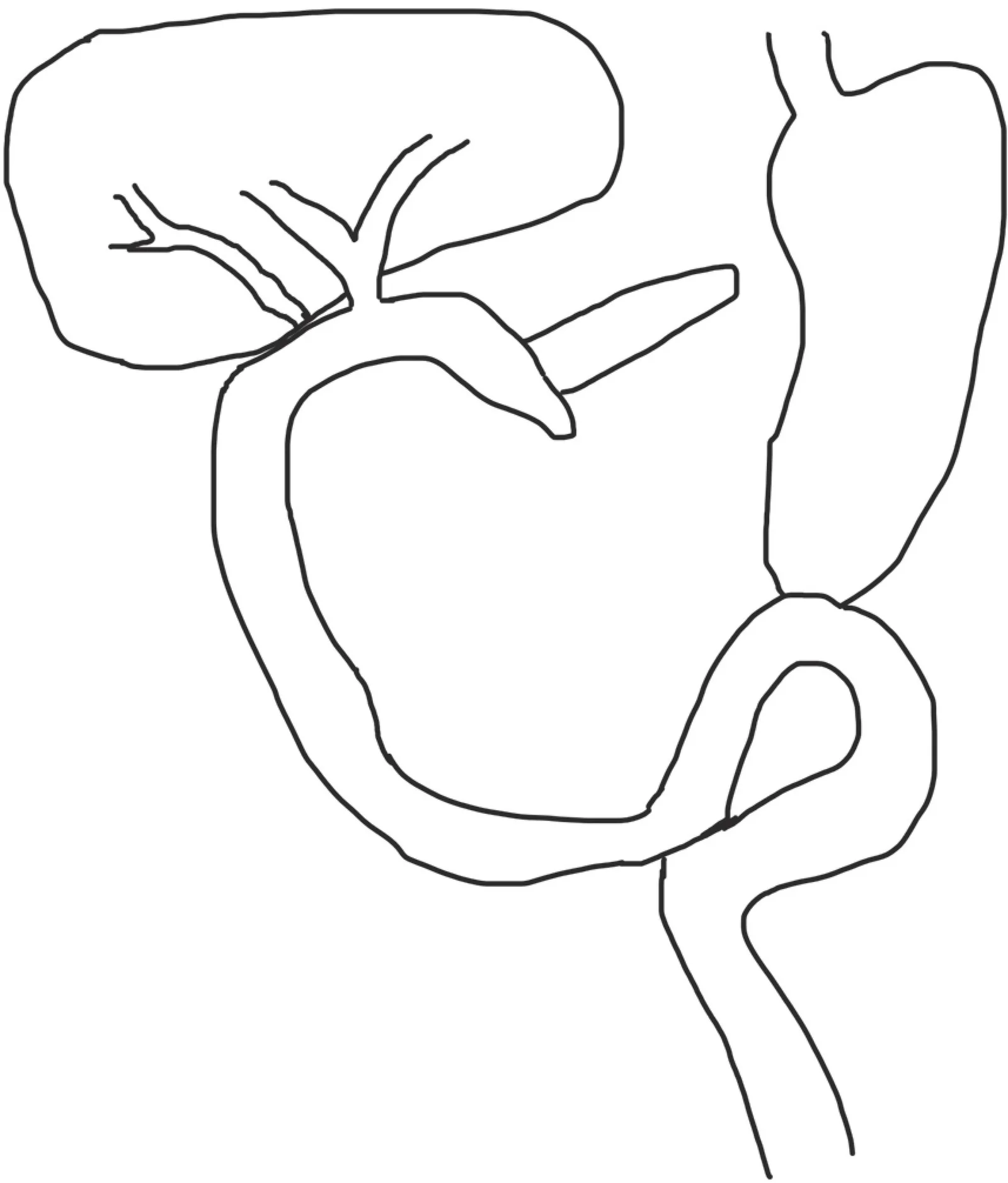
Schematic illustration of the surgical anatomy and complete obstruction of the right posterior sectoral bile duct after pancreatoduodenectomy.

**Fig. 3:**
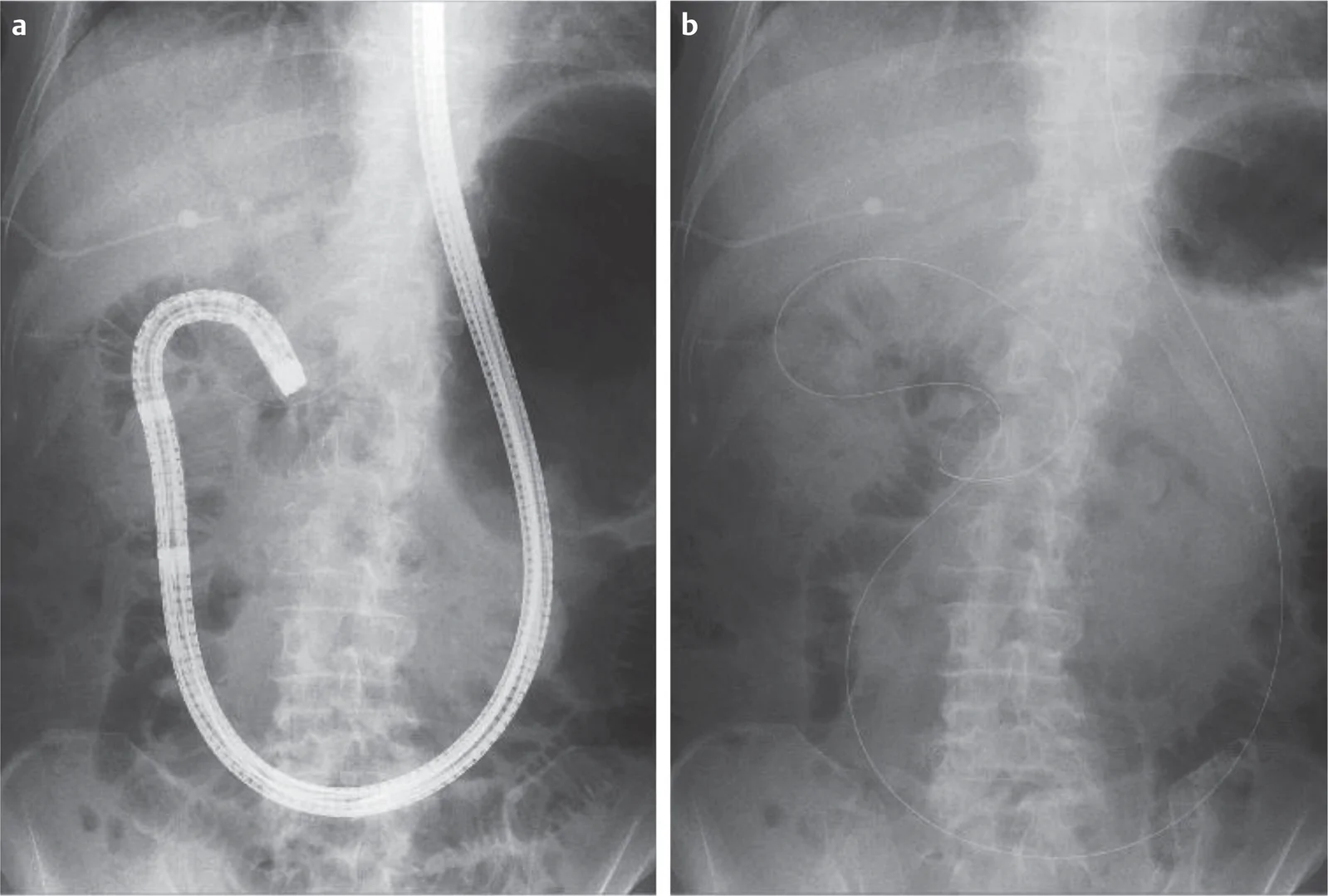
( **a** ) Fluoroscopy showed the colonoscope reaching the deepest part of the afferent limb. ( **b** ) After withdrawal of the colonoscope, the guidewire remained in place at the deepest part of the afferent limb.

**Fig. 4:**
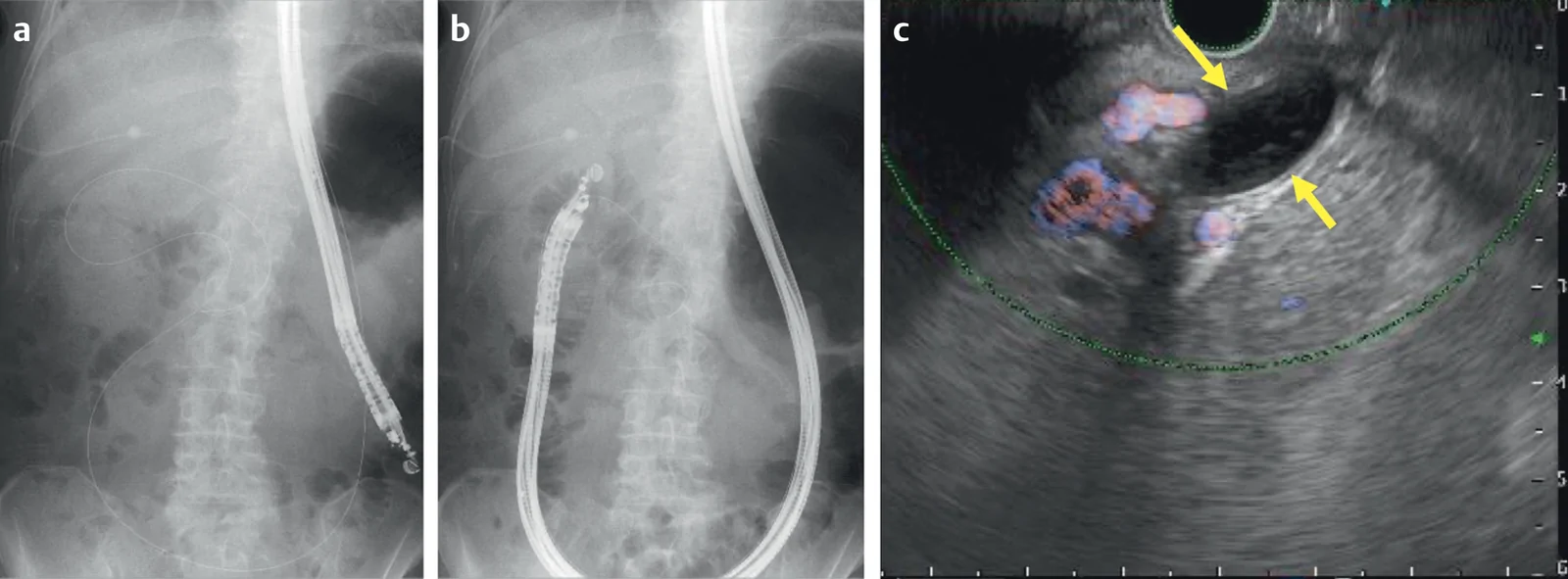
( **a** ) Fluoroscopy showing the advancement of the oblique-viewing linear-array echoendoscope along the guidewire. ( **b** ) The echoendoscope reached the jejunum adjacent to the obstructed bile duct. ( **c** ) Endoscopic ultrasonography visualized the obstructed and dilated bile duct (arrow) adjacent to the jejunum.

**Fig. 5:**
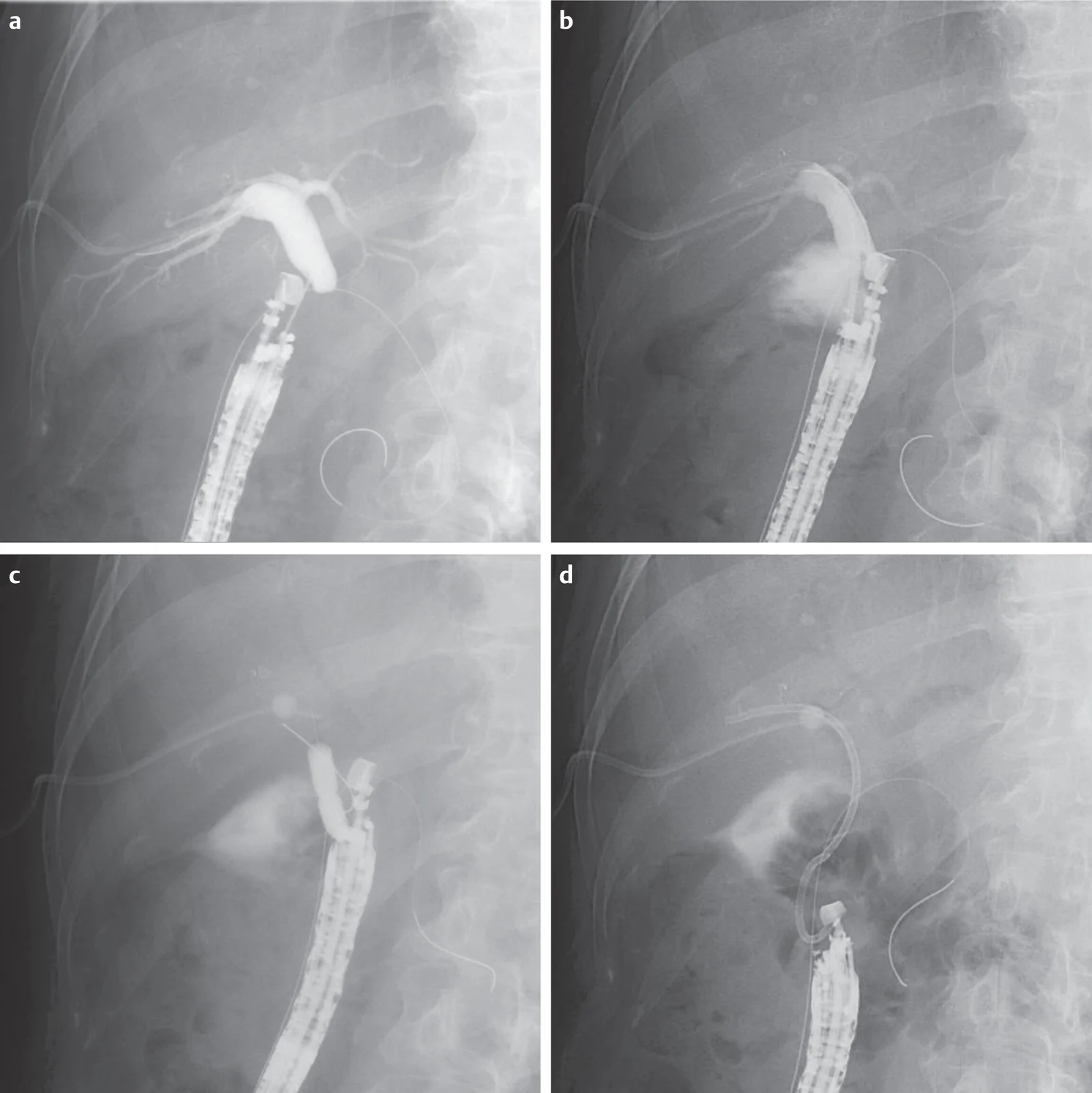
( **a** ) Puncture of the bile duct with a 19-gauge needle and cholangiography confirming the isolated posterior bile duct. ( **b** ) Tract dilation using a drill-type dilator. ( **c** ) Balloon dilation of the puncture tract. ( **d** ) Placement of a 7-Fr, 10-cm plastic stent between the posterior bile duct and the jejunum.

**Video 1** Guidewire-assisted insertion of an oblique-viewing linear-array echoendoscope followed by transjejunal endoscopic ultrasound–guided biliary drainage.


No case of EUS-BD using an oblique-viewing linear-array echoendoscope in patients with surgically altered anatomy has been reported. The colonoscope was used to confirm the feasibility of deep enteral access before echoendoscope insertion and to place a guidewire as a visual landmark for the controlled advancement of the echoendoscope. This two-step approach may facilitate EUS-BD using routinely available endoscopic devices.

Endoscopy_UCTN_Code_TTT_1AS_2AD

## Declaration of GenAI Use

The authors confirm that no generative artificial intelligence was used in the preparation of this manuscript.
